# Measuring researchers’ potential scholarly impact with structural variations: Four types of researchers in information science (1979–2018)

**DOI:** 10.1371/journal.pone.0234347

**Published:** 2020-06-22

**Authors:** Jianhua Hou, Xiucai Yang, Chaomei Chen

**Affiliations:** 1 School of Information Management, Sun Yat-sen University, Panyu District, Guangzhou, Guangdong, China; 2 College of Economics and Management, Dalian University, Dalian Economic Technological Development Zone, Dalian, China; 3 College of Computing and Informatics, Drexel University, Philadelphia, PA, United States of America; 4 Department of Information Science, Yonsei University, Seoul, Republic of Korea; KU Leuven, BELGIUM

## Abstract

We propose a method to measure the potential scholarly impact of researchers based on network structural variations they introduced to the underlying author co-citation network of their field. We applied the method to the information science field based on 91,978 papers published between 1979 and 2018 from the Web of Science. We divided the entire period into eight consecutive intervals and measured structural variation change rates (ΔM) of individual authors in corresponding author co-citation networks. Four types of researchers are identified in terms of temporal dynamics of their potential scholarly impact—1) Increasing, 2) Decreasing, 3) Sustained, and 4) Transient. The study contributes to the understanding of how researchers’ scholarly impact might evolve in a broad context of the corresponding research community. Specifically, this study illustrated a crucial role played by structural variation metrics in measuring and explaining the potential scholarly impact of a researcher. This method based on the structural variation analysis offers a theoretical framework and a practical platform to analyze the potential scholarly impact of researchers and their specific contributions.

## 1 Introduction

The research impact of scientists has always been a major topic, especially in information science (IS) and scientometrics. Extensively known quantitative measures of researchers’ scholarly impact primarily include indicators such as the number of publications, number of co-authors, citation frequency, and h-index [1]. Moreover, a researcher’s scholarly impact is analyzed in terms of properties from co-authorship networks or co-citation networks (e.g., centrality)[2]. In addition, researchers have proposed integrating various indicators from multiple perspectives [3–8].

In IS, several studies have investigated the scholarly impact of researchers from the perspective of citation network analysis. For example, White et al. used the author co-citation analysis (ACA) approach to measure the influence of 39 authors in the IS field [9]. Moreover, follow-up researchers used ACA or co-author methods to analyze researchers in the IS domain and identify researchers with scholarly impact in this field by combining scientometrics indicators.

Recent years have witnessed an upsurge in the publication of papers in the IS domain. Meanwhile, new knowledge and topics are constantly emerging in this field, and new researchers with potential scholarly impact on the field have also emerged. The impact of the introduction of new knowledge on the fundamental network structure of existing research fields is a crucial aspect of the development of scientific activities. A major form of creative work is to bridge previously disjoint bodies of knowledge [10–13]. Chen proposed a predictive analytic model—structural variation analysis (SVA) [14]. The SVA model focuses on structural variations of underlying networks by transformative connections introduced in new publications over time and measures the transformative potential of a scholarly publication, which provides a promising analytical and explanatory method that can be applied to the study of researchers’ potential scholarly impact.

Based on the SVA framework, this paper focuses on measuring the potential scholarly impact of researchers and categorizing them in terms of the dynamics of structural variation metrics. We define four types of researchers in terms of their structural variation patterns with the IS field as an example. The SVA-based approach has a unique advantage of linking researchers’ specific contributions to their scholarly impact indicators because these reflect the extent to which researchers’ specific publications bring emergent changes to the underlying networks of the knowledge domain in question. Major contributions of this study include the following:

1. Based on the SVA, researchers with potential scholarly impact in the IS domain are measured and identified in different time periods. This study provides a theory-driven analytics platform to analyze the potential scholarly impact of researchers and their specific contributions.
2. This study characterizes four types of the potential scholarly impact based on how their structural variation metrics change over time, revealing that the ΔM within each type of researchers was proportional to the researchers’ potential scholarly impact.
3. This study reveals the position and structural features of different types of researchers with potential scholarly impact in co-author networks. SVA plays a crucial role in measuring and explaining the potential scholarly impact of a researcher.

## 2 Related work

To date, several studies have focused on exploring the academic impact of individual research in bibliometrics and scientometrics. The related research mainly focuses on the analysis of an author’s influence based on structural properties of citation networks and the analysis of an author’s influence based on quantitative statistical indicators.

### 2.1 Co-author network analysis

Lately, a growing degree of attention has been paid to researchers’ scholarly impact through the co-author network analysis or citation network analysis. For example, the impact of researchers in cooperation networks is analyzed through citation network indicators, such as degree centrality, closeness centrality, and betweenness centrality, which are used to measure researchers’ impact in collaboration networks [15]. Initially, some studies ascertained whether the structure of the citation network predicts future citations [16], and a recent approach started leveraging citation network information available at publication time to anticipate a paper’s future impact [14,17].

Collaboration is considered having positive effects on researchers’ performance by enabling the exchange of resources, knowledge, and experience [18]. Several researchers have applied social network analysis (SNA) of the collaboration network to detect academic impact [19–21]. Studies have repeatedly found a positive correlation between collaboration and productivity [22–29]. Scientific cooperation can enhance the performance of the research output. The higher the productivity of a researcher, the greater its impact; however, it also depends on the mode of cooperation, motivation of cooperation, and position of researchers in the cooperative network. In some cases, collaboration even exerts a negative impact on productivity [30].

Scientific collaboration networks are well connected [31], and the scientific communities seemingly constitute a small world [32]. The influence of an author on the cooperative network can be revealed. Usually, the higher the cooperation frequency of an author in the cooperative network, the higher the density of forming the small-world network, and the greater the influence of the author in the cooperative network. For most authors, the bulk of the paths between them and other scientists in the network go through just one or two of their collaborators [33]. In addition, Contandriopoulos et al. examined the correlation between the position of researchers in the cooperative network structure and the influence of researchers [34]. Researchers who occupy a bridging position in a network are more likely to exhibit a higher publication performance and influence than those connected mostly with the same small group of researchers in all their activities. Contandriopoulos et al. suggested that some of the most bridging and highest-performing researchers are not necessarily at the core of the network and rely more on external collaborations [34].

The network structure, and the positions of researchers in the network in particular, may correlate with the influence of researchers. Different network structures and the positions of researchers in a collaborative network are crucial to assess the influence of researchers. However, the impact of the change in the network structure on researchers, especially the change of new researchers on the existing cooperative network structure, will be a crucial and novel method to measure the potential influence of researchers.

The co-author network analysis reveals the impact of author collaboration on the output of researchers, whereas the author citation analysis reveals the scholarly impact of researchers; both are critical methods to measure the impact of researchers in the IS field currently, and some studies, in fact, have combined both methods for analysis. Usually, a positive correlation exists between author cooperation or author cited and author’s scholarly impact [15,35]. However, all these studies are based on the baseline knowledge networks (e.g., author cooperation network and co-citation network), which analyze the existing network structure and do not consider the change in the network structure on the dynamic change of researchers’ influence.

### 2.2 Statistical analysis

Statistical analysis is a simple and effective method to analyze the influence of an author through quantitative statistical indicators. The method has led to various indicators such as the number of refereed journal papers published and citations received, and measures in the form of an impact factor [36], h-index[1], g-index [37], and also altmetrics [38].

#### 2.2.1 Bibliometric indicators

The influence of researchers or papers has been mainly measured by citation counts, including total citations, h-index, and citations per publication [1,3,37,39–43]. However, the extent to which these indicators measure scholarly impact remains unclear. More importantly, these indicators convey little or no information regarding the context of a scholarly impact.

Ajiferuke & Wolframproposed an idea of citer (unique individuals who have cited a given author) analysis to assess the author’s reach or influence in a field; they found that citer counts analysis offer better results than those based on more traditional citation counts for differences in author assessments [44]. Using the statistical index analysis to measure the author’s influence is a simple measurement method. However, these measures cannot precisely and effectively analyze the real influence of the author in their research field. For example, given the diversity of citation motivation, there are lengthy debates on assessing the influence of papers or authors with traditional indicators [45–51], including the statistical sources and counting methods of citation counts [44,52–55], whether the citation can reflect the research contributions of the paper [45,56–59], as well as the subjectivity of the author of the citation and the common issues of self-citation [60–64].

The studies identified above are based on traditional bibliometrics and have assessed the influence of authors from the perspective of statistics. However, an author’s position in a citation network or collaborative network can be usefully exploited. This study analyzes the influence of an author from the perspective of the changes associated with the structure of an underlying network. Thus, we expect that we will be able to characterize a research’s influence more accurately and more specifically.

#### 2.2.2 Altmetrics

Recent years have encountered a growing number of studies on the author’s influence through altmetrics, including new analysis software. Altmetrics are non-traditional metrics that cover not just citation counts but also downloads, social media shares, and other measures of the impact of research outputs [65]. Altmetrics measures the broader impact of research on society [5,7,8,66–70], especially by using a much wider set of resources, including social media posts, press releases, news articles, and political debates stemming from academic work, as well as assesses wider non-academic impact [6]. In 2014, Bornmannanalyzed the advantages and disadvantages of measuring the impact of using altmetrics [4].

Altmetrics offers several advantages to analyze the author’s influence. For example, the evaluation of author’s influence through altmetrics indicator offers the advantage of immediacy owing to the rapid and efficient dissemination of research results on social media, enabling more subject researchers and the public to quickly focus on the research results of researchers through social media. Thus, the evaluation of authors’ influence based on the social media indicators has the characteristics of interdisciplinary and more extensive advantages. However, several problems exist in the evaluation based on altmetrics. Researchers are concerned about data collection, data manipulation, platform stability, and other issues of altmetrics indicator, and it remains unclear whether altmetrics indicators can really capture or reflect scholars’ social influence [65]. Furthermore, altmetrics indicators based on social media are the main evaluators of the authors’ “social influence,” and it is challenging to reflect the authors’ “academic influence.”

Previous studies have primarily analyzed researchers’ scholarly impact from a citation perspective using quantitative indicators of citation or structural information of citation (cooperative) network. The researchers’ scholarly impact has rarely been studied from the perspective of newly incoming authors (articles) on their basic knowledge infrastructure (citation network structure).

In 2012, Chen proposed a theoretical and computational model that predicts the transformative potential of a scientific publication in terms of the extent to which it profoundly alters the intellectual structure of the state of the art [14]. This model is called the SVA, which primarily focused on novel boundary-spanning connections introduced by a new article to the intellectual space and by using the boundary-spanning effect to estimate the potential impact of contributing literature. Chen tested the impact of structural variations on cases from five different fields of research and found statistically significant predictive powers in three of them, suggesting that this is a promising computational and explanatory approach to elucidate the research impact [14]. SVA is available in CiteSpace and has been used in more recent studies of the potential structure variation in a field, for example, science mapping [17]. However, this is the first study to adopt SVA to examine researchers as opposed to scholarly publications. As a unit of the analysis, researchers are at a higher level of granularity because one research may be related to a growing set of publications.

In the following sections, we will investigate the potential scholarly impact of the IS researchers who were divided into four types, from the perspective of incoming authors’ or articles’ impact on the basic citation network structure. Next, we will examine the correlation among the potential scholarly impact of different types of researchers, the author co-citation frequency, the number of papers published in this field, and the location and the structural features in the co-author network.

## 3 Data and methods

In this study, we applied SVA to measure the potential scholarly impact of researchers in the IS field. In particular, the modularity change rate index (ΔM) and researchers’ position and structural properties in the network are utilized. We retrieved bibliographic records in the field of IS form the Web of Science. We visualized and analyzed the dataset with CiteSpace (version 5.3.R4 SE) [17].

### 3.1 Data collection

**Fig 1:**
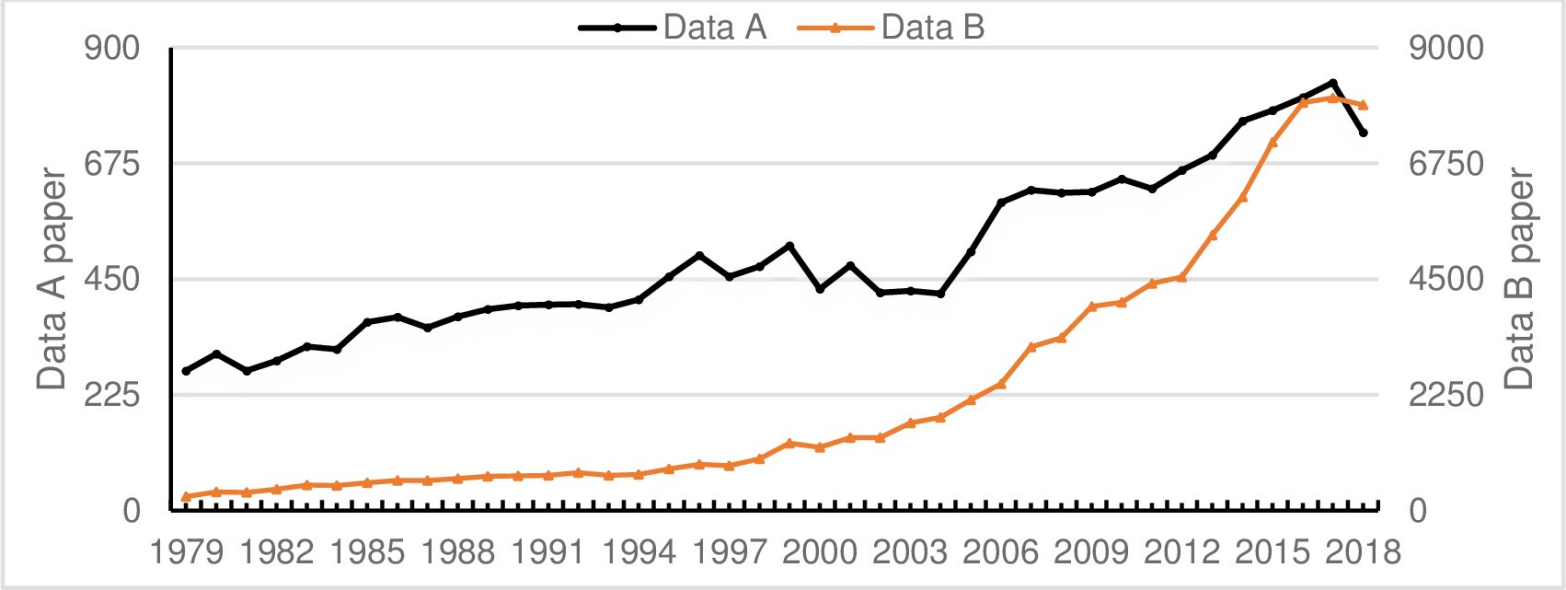
Sets A and B number of documents changes over time.

In this study, we adopted the citation expansion method [71] to collect the relevant articles. First, we selected a set of seed journals as the initial set. Then, using citation expansion, we retrieved all references that cited the initial set for the subsequent analysis (Fig 1). In the previous analysis and definition of the IS field research evolution and research front, several studies in the IS field adopted a collection of 12 journals (S1 Appendix) as a representative body of the relevant literature [72–82]. However, the selected journals might not necessarily and sufficiently represent the IS field [83]. Compared with the method of journal-based data collection, the data obtained by citation expansion were more comprehensive and relevant in terms of its coverage. Our methods were as follows:

Step 1: Determine the initial journal collection, Set A.

We selected the following five journals as our initial journal collection: “Information Processing & Management” (5-year impact factor: 3.295); “JASIS/JASIST” (3.101); “Journal of Documentation” (1.601); “Journal of Information Science” (2.155); and “Scientometrics” (2.71). Fig 1 shows the year-by-year distribution of data collected from 1979 to 2018.

Step 2: Determine the journal collection used in this study, Set B.

In the core collection of the Web of Science, data of each journal in Set A from 1979 to 2018 were retrieved respectively. The citation reporting function in the Web of Science was used to identify all papers that cited the initial set. Then, we combined the initial set and extended set data, and eliminated the duplicate data; the dataset was called Set B. Set B contains 91,978 records, which are analyzed in this study; Fig 1 shows their annual distribution. Using these data, we examined the evolution of two types of networks—ACA networks and co-author networks. Of note, the expanded set provides a broader context of the core IS literature.

### 3.2 Structural variation analysis (SVA)

To measure the influence of a researcher’s scholarly work, a fundamental concept is the role of the researcher or his/her work in spanning and bridging otherwise disjoint bodies of the existing knowledge. SVA in citation networks was proposed by Chen [14]; it measures the change in a network introduced by a new paper or a researcher and then ranks researchers according to the metric. For scientists, such boundary-spanning connections contribute to the knowledge of the field in question and it is seen as the scholarly influence of scientists. The theoretical underpinning of the structural variation is that scientific discoveries, to a considerable extent, can be conceptualized as the consequences of boundary spanning, brokerage, and synthesis mechanisms in an intellectual space [84]. The basic assumption in the structural variation approach is that the extent of a departure from the current intellectual structure is a necessary condition for a potentially transformative idea in science [14].

SVA includes three primary structural variation metrics, namely, modularity change rate (MCR) ΔM, inter-cluster linkage (CL), and centrality divergence (C_KL_). The ΔM index tracks the difference between the structure of an existing network and newly added connections that would change the modularity of the current network most.

ΔM measures the structural changes of the underlying network induced by connections contributed by new publications. More specifically, ΔM measures how the structure of a network changes at the cluster level. For example, two previously separated groups of authors may become increasingly interwoven and form a larger group with members from both previously identified groups. For more details, see [14].

The higher the value of the ΔM index, the greater the potential impact of new papers or authors is expected to have on the network as a whole. Both ΔM and CL focus on the impact of adding new connections that would alter the network structure substantially. However, ΔM measures the updated modularity, which may increase and decrease, depending on the network structure and where the new links are distributed. If new papers or authors add new links within a cluster of the current network, these links will reinforce the existing structure and increase the overall modularity. If new links connect distinct clusters, the modularity of the network will be reduced. CL focuses on the effect of between-cluster links before and after a new paper becomes available. C_KL_ measures the structural variations arising from a new article based on the divergence of the distributions of the betweenness centrality measures of all the nodes in the network before and after information from the new article is taken into account.

In this study, we adopted ΔM as a measure of the potential scholarly impact of a research. A co-authorship network depicts patterns of collaboration within the academic community [85]. ΔM associated with such networks denotes the structural changes in these networks. Suppose the co-authorship network *G* is partitioned by a partition *C* into *k* clusters such that *G* = *c*_1_ + *c*_2_ + … + *c*_*k*_, Q(*G*) is defined as follows, where *m* is the total number of edges in the network *G*, *n* is the number of nodes in *G*. *δ*(*c*_*i*_, *c*_*j*_) is known as the Kronecker’s delta; it is 1 if nodes *n*_*i*_ and *n*_*j*_ belong to the same cluster and 0 otherwise. Furthermore, deg(*n*_*i*_) is the degree of node *n*_*i*_. The range of Q(*G*) is between –1 and 1.

$$Q(G,C)=\frac{1}{2m}\sum_{i,j=0}^{n}\delta (c_{i},c_{j})∙(A_{ij}-\frac{deg(n_{i})∙deg(n_{j})}{2m})$$ (1)

The modularity of a network is a measure of the overall network structure; its range is between –1 and 1. The MCR of a scientific paper measures the relative structural change because of the information from the published paper with reference to a baseline network. For each article a, and a baseline network *G*_baseline_, we defined the MCR as follows:

$$MCR(a)=\frac{Q(G_{baseline},C)-Q(G_{baseline}⊕G_{a},C)}{Q(G_{baseline},C)}∙100$$ (2)

Where *G*_baseline_ and *G*_a_ are the updated baseline network by information from article a. For example, suppose reference nodes *n*_*i*_ and *n*_*j*_ are not connected in a baseline network of co-cited references but are co-cited by article a, a new link between *n*_*i*_ and *n*_*j*_ will be added to the baseline network. This way, the article changes the structure of the baseline network.

CiteSpace is an information visualization software system suitable for multivariate, time-sharing, and dynamic complex network analysis [11,17,72,86,87]; it takes a set of bibliographic records as its input and models the intellectual structure of the underlying domain in terms of a synthesized network based on a time series of networks derived from each year’s publications. CiteSpace supports several types of bibliometrics studies, including collaboration network analysis, co-word analysis, ACA, document co-citation analysis, text and geospatial visualizations. The SVA function is available in CiteSpace and can be used along with any type of the networks mentioned above. In this study, we conducted an SVA on author co-citation networks between 1979 and 2018.

### 3.3 Four types of researchers

**Fig 2:**
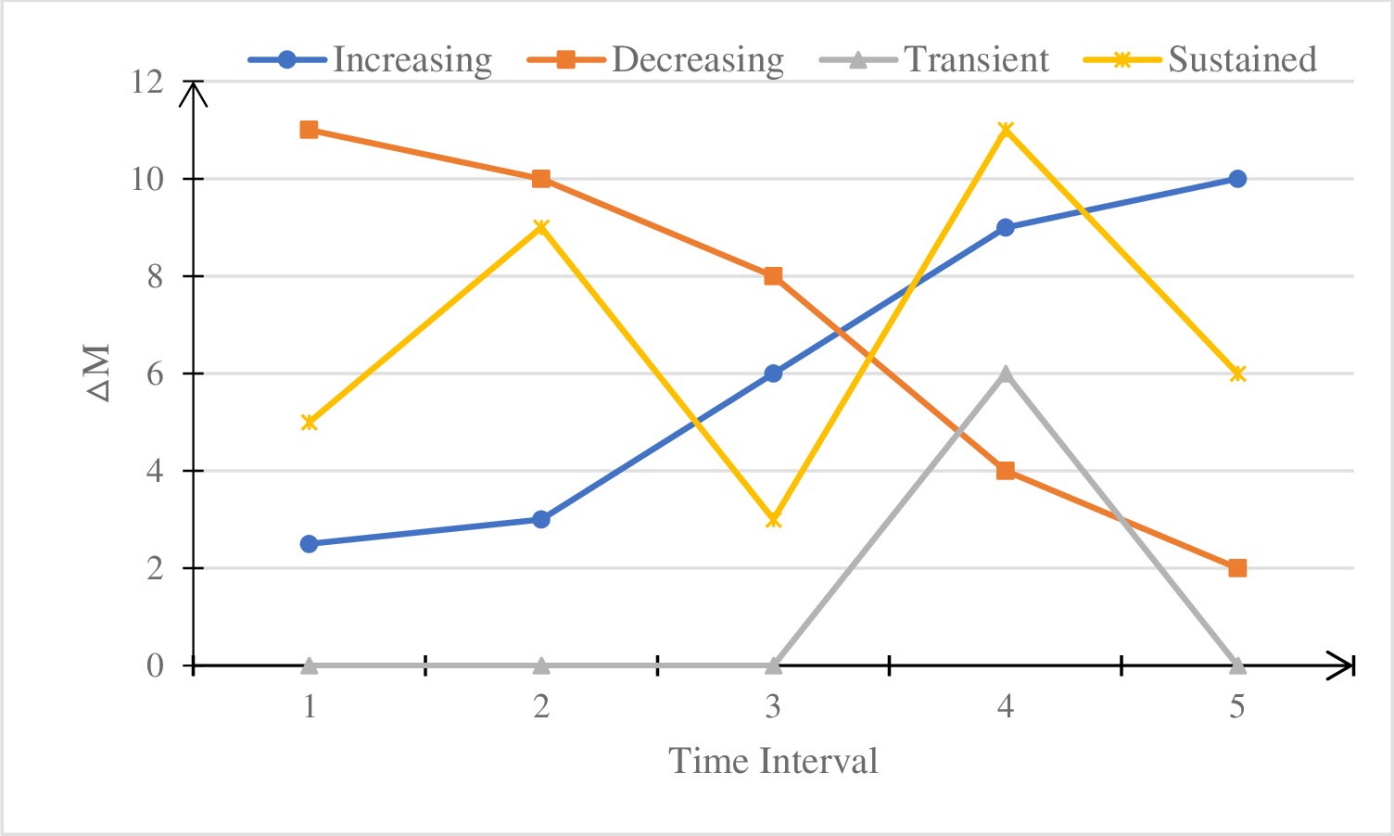
Trajectory of ΔM values of four types of researchers in different time periods.

In this study, we characterized researchers into four types of scholarly impact potential based on their ΔM variation patterns. Researchers with monotonically increasing or decreasing ΔM values are defined as the increasing and decreasing types, respectively. The other two types of researchers are sustained and transient for researchers whose ΔM values either fluctuate over multiple years or become non-zero only once or twice. ΔM_i_ denotes a researcher’s ΔM in the *i*th interval, which is the difference between the modularity of the network from the *i*th interval and the modularity of the network from the (i-1)th interval (Fig 2). In Fig 2, to highlight the trajectories of ΔM of transient researchers, we set 0 for the time interval when ΔM = 0.

1. Increasing (↑): If a researcher’s ΔM_i_< = ΔM_j_, for *i* < *j*, during his/her research period, the researcher is considered to be an increasing type.
  The increasing researcher’s ΔM variation range = ΔM_j_–ΔM_i_.
2. Decreasing (↓): If a researcher’s ΔM_i_> = ΔM_j_, for *i* < *j*, during his/her research period, the researcher is defined as a decreasing researcher.
  The decreasing researcher’s ΔM variation range = ΔM_i_–ΔM_j_.
3. Transient (–): If a scholar has only one ΔM > 0 in his/her research period, that is, a scholar has only one ΔM_k_ > 0, for *i* = *k*, otherwise ΔM_i_ = 0, the researcher is called the transient researcher.
  The transient researcher’s ΔM variation range = ΔM_k_.
4. Sustained (≈): If a scholar has ΔM > 0 in multiple intervals but the pattern does not meet the conditions of (1), (2), or (3), the researcher is called a sustained researcher.
  The sustained researcher’s ΔM variation range = sqrt(((ΔM_1_–$\bar{\Delta }M$)^2^ +(ΔM_2_–$\bar{\Delta }M$)^2^+……(ΔMn–$\bar{\Delta }M$)^2^)/*n*).

## 4 Results

To detect the characteristics of potential influential researchers in the field of IS, we identified researchers with potential influence in the IS field in different time periods from 1979 to 2018 based on the value of ΔM by SVA. Then four types of researchers are identified based on the change in ΔM value of different researchers in different time intervals, and the general characteristics of the change in ΔM value of four types of researchers are further explored. Based on the four types of researchers identified, we studied the location characteristics of different types of researchers in the co-authorship network and further explored the impact of change in time interval on different types of researchers.

### 4.1 Computing the modularity change rate of researchers

To predict researchers’ scholarly impact in IS research, we divided Set B from 1979 to 2018 into eight time intervals. We conducted SVA on the author co-citation network of each period and identified researchers with high potential scholarly impact in each period. We used CiteSpace to calculate the ΔM values of each researcher included in Set B. Tables 1 and 2 list the top 20 researchers with the largest ΔM values for each period 1979–1998 and 1999–2018, respectively.

**Table 1:** Top 20 researchers with the largest ΔM in each of the 5-year time intervals (1979–1998).

1979–1983	ΔM	1984–1988	ΔM	1989–1993	ΔM	1994–1998	ΔM
Smith L C	21.17	Vlachy J	25.48	Efthimiadis E N	21.19	Ingwersen P	24.96
Hawkins D T	15.93	Fox E A	18.79	Luukkonen T	19.37	Hjorland B	22.19
Schrader A M	12.24	Schwartz C	16.12	Harter S P	16.82	Kishida K	21.21
Garfield E	9.54	Kinnucan M T	15.43	Leydesdorff L	15.23	Sugar W	19.16
Salton G	7.08	Dervin B	15.31	Shaw W M	12.86	Spink A	18.54
Yablonsky A I	6.55	Jarvelin K	13.3	Baker D R	12.85	Buckland M K	18.48
Mulkay M	6.29	Schubert A	13.03	Doszkocs T E	10.86	Cronin B	11.86
Buell D A	6.21	Pierce S J	10.76	Swanson D R	8.76	Harter S P	11.83
Knorr K D	5.28	Mccain K W	10.05	Koenig M E D	8.3	Hoerman H L	11.74
Keren C	5.26	Salton G	10.02	Carley K	7.57	Chen H C	11.3
Fugmann R	4.55	Belkin N J	9.91	Schuegraf E J	7.54	Losee R M	11.09
Shinebourne J	4.28	Vinkler P	9.32	Bayer A E	7.07	Gluck M	9.4
Bates M J	4.08	Pravdic N	8.99	Bates M J	6.8	Cortez E M	8.26
Magrill R M	3.39	Chubin D E	8.21	Sengupta I N	6.29	Campanario J M	7.94
Vlachy J	3.24	Macroberts M H	8.1	Braun T	5.92	Davenport E	7.7
Walker D E	3.22	Smith L C	7.71	Hersh W R	5.57	Wang P L	7.67
Mccain K W	3.13	Bates M J	7.46	Salton G	5.53	Borgman C L	6.97
Travis I L	3.03	Tiamiyu M A	7.41	Thompson P	5.46	Hersh W R	6.81
Mcgarry K	2.94	Rice R E	6.58	Plomp R	5.31	Miquel J F	6.09
Hubert J	2.82	Case D	6	Larson R	5.27	Rajashekar T B	5.96

**Table 2:** Top 20 researchers with the largest ΔM in each of the 5-year time intervals (1999–2018).

1999–2003	ΔM	2004–2008	ΔM	2009–2013	ΔM	2014–2018	ΔM
Ding Y	40.01	Thelwall M	73.99	Leydesdorff L	59.68	Thelwall M	113.62
Kobayashi M	27.33	Jansen B J	62.96	Bornmann L	35.2	Mingers J	70.17
Borgman C L	23.04	Yang K D	44.52	Kurtz M J	33.42	Abramo G	51.03
Song M	20.49	Balinski J	33.32	Chen C M	30.87	Zitt M	48.71
Borlund P	18.72	Kostoff R N	23.03	Ahlgren P	29.33	Serenko A	48.39
Hjorland B	18.49	Feng L	15.37	Thelwall M	29.0	Guan J	46.98
Efthimiadis E N	16.64	Fan W G	14.75	Franceschini F	28.37	Bornmann L	44.09
Dominich S	14.97	Price L	14.6	Perc M	26.85	Ebadi A	40.94
Cole C	11.9	Chau M	14.47	Rafols I	25.17	Haustein S	40.3
Thelwall M	11.6	White R W	13.44	Jansen B J	24.49	Leydesdorff L	39.09
Beaulieu M	11.03	Freeman R T	12.66	Egghe L	23.42	Zupic I	36.83
Egghe L	10.03	Lin T Y	12.4	Persson O	22.65	Yan E	34.19
Davenport E	9.82	Pant G	11.32	Li J	22.63	Huang M	33.59
Bar-Ilan J	9.47	Scharnhorst A	11.22	Franceschet M	19.15	Fairclough R	33
Carpineto C	9.27	Topi H	10.35	Dolfsma W	18.68	Cimenler O	30.7
Cronin B	8.92	Cronin B	10.25	Vieira P C	18.61	Zhu Y	30.62
Chen C M	8.72	Mathassan M	9.46	Upham S P	18.6	Gallivan M	29.99
White H D	7.94	Zhu B	8.76	Hicks C	18.38	Sriwannawit P	27.19
Greisdorf H	7.54	Bar-Ilan J	8.72	Yoon B	18.32	Bordons M	26.48
Toms E G	6.88	Wallin J A	8.72	Zhang Lin	18.25	Kim Y	26.14

During 1979–2018, researchers with the highest ΔM values includes Smith L C (21.17) in 1979–1983, Vlachy J (25.48) in 1984–1998, Efthimiadis E N (21.19) in 1989–1993, Ingwersen P (24.96) in 1994–1998, Ding Y(40.01) in 1999–2003, Thelwall M (73.99) in 2004–2008, Leydesdorff L (59.68) in 2009–2013, and Thelwall M (113.62) in 2014–2018.

Several researchers appeared multiple intervals in the eight periods, but how their ΔM values change varied considerably. For example, Garfield E.’s ΔM values gradually decreased. In 1979–1983, his ΔM value was 9.54. In 1984–1988, it declined to 5.67. In 1989–1993, it decreased further to 1.01. In 1994–1998, it became 0.72, and in the most recent period of 2004–2008, it was 0.13.

In contrast, Leydesdorff L’s ΔM value fluctuated. In 1984–1988, Leydesdorff L’s ΔM value was 1.39. In 1989–1993, it increased to 15.23. In 1994–1998, it dropped to 1.07. In 1999–2003, it was 5.69, in 2004–2008 was 7.93. In 2009–2013, it raised again to 59.68, and in 2014–2018 was 39.09.

Yan E is an example of a researcher with a steady trend of increase over time. It increased from 0.18, to 9.52, and 34.19 in 2004–2008, 2009–2013, and 2014–2018, respectively.

Some researchers might only have a non-zero ΔM value once throughout the eight time windows. For example, Glenisson P, Cimenler O, and Park I Glenisson P appeared once in 2004–2008 with a ΔM of 7.52. Cimenler O appeared in 2014–2018 with a ΔM of 30.7. Park I appeared in 2014–2018 with a ΔM of 23.99.

### 4.2 The distribution of ΔM

To analyze the correlation between the dynamics of a researcher’ ΔM values and the researcher’ potential scholarly impact, we characterized researchers into four types based on their ΔM variation patterns. Table 3 lists researchers in these four types, including the top 10 researchers with the largest changes of ΔM. Table 4 lists the number of times each of the four types of researchers has appeared in the eight time periods.

**Table 3:** Top 10 researchers of each of the four types and the ranges of their ΔMs.

Increasing (↑)		Decreasing (↓)		Sustained (≈)		Transient (-)	
Name	Range of ΔM	Name	Range of ΔM	Name	Range of ΔM	Name	Range of ΔM
Mingers J	70.07	Franceschini F	27.34	Thelwall M	45.97	Yang KD	44.52
Zitt M	48.52	Luukkonen T	18.8	Jansen B J	29.81	Ebadi A	40.94
Abramo G	48.38	Zhang Lin	16.84	Ding Y	18.64	Zupic I	36.83
Serenko A	48.22	Milojevic S	14.99	Chen C M	13.05	Kurtz M J	33.42
Haustein S	39.43	Kinnucan M T	14.65	Hjorland B	10.17	Balinski J	33.32
Bornmann L	39.22	Hawkins D T	13.93	Borgman C L	9.25	Fairclough R	33
Yan E	34.01	Smith L C	13.46	Fox E A	8.65	Cimenler O	30.7
Perc M	25.85	Pierce S J	10.04	Ingwersen P	8.43	Gallivan M	29.99
Rafols I	24.84	Garfield E	9.41	Egghe L	8.33	Sriwannawit P	27.19
Rotolo D	22.92	Dominich S	7.2	Efthimiadis E N	8.31	Armando Ronda-Pupo Guillermo	25.96

**Table 4:** Top 10 researchers of each type and, #ΔM, the number of intervals in which they have a non-zero ΔM.

Increasing (↑)		Decreasing (↓)		Sustained (≈)		Transient (-)	
Name	#ΔM	Name	#ΔM	Name	#ΔM	Name	#ΔM
Zitt M	6	Garfield E	4	Ingwersen P	8	Yang KD	1
Mingers J	4	Franceschini F	2	Egghe L	6	Ebadi A	1
Serenko A	3	Luukkonen T	2	Chen C M	5	Zupic I	1
Bornmann L	3	Zhang Lin	2	Hjorland B	5	Kurtz M J	1
Yan E	3	Milojevic S	2	Borgman C L	5	Balinski J	1
Abramo G	2	Kinnucan M T	2	Thelwall M	4	Fairclough R	1
Haustein S	2	Hawkins D T	2	Jansen B J	3	Cimenler O	1
Perc M	2	Smith L C	2	Ding Y	3	Gallivan M	1
Rafols I	2	Pierce S J	2	Fox E A	3	Sriwannawit P	1
Rotolo D	2	Dominich S	2	Efthimiadis E N	3	Armando Ronda-Pupo Guillermo	1

#### Type A: The increasing type

**Fig 3:**
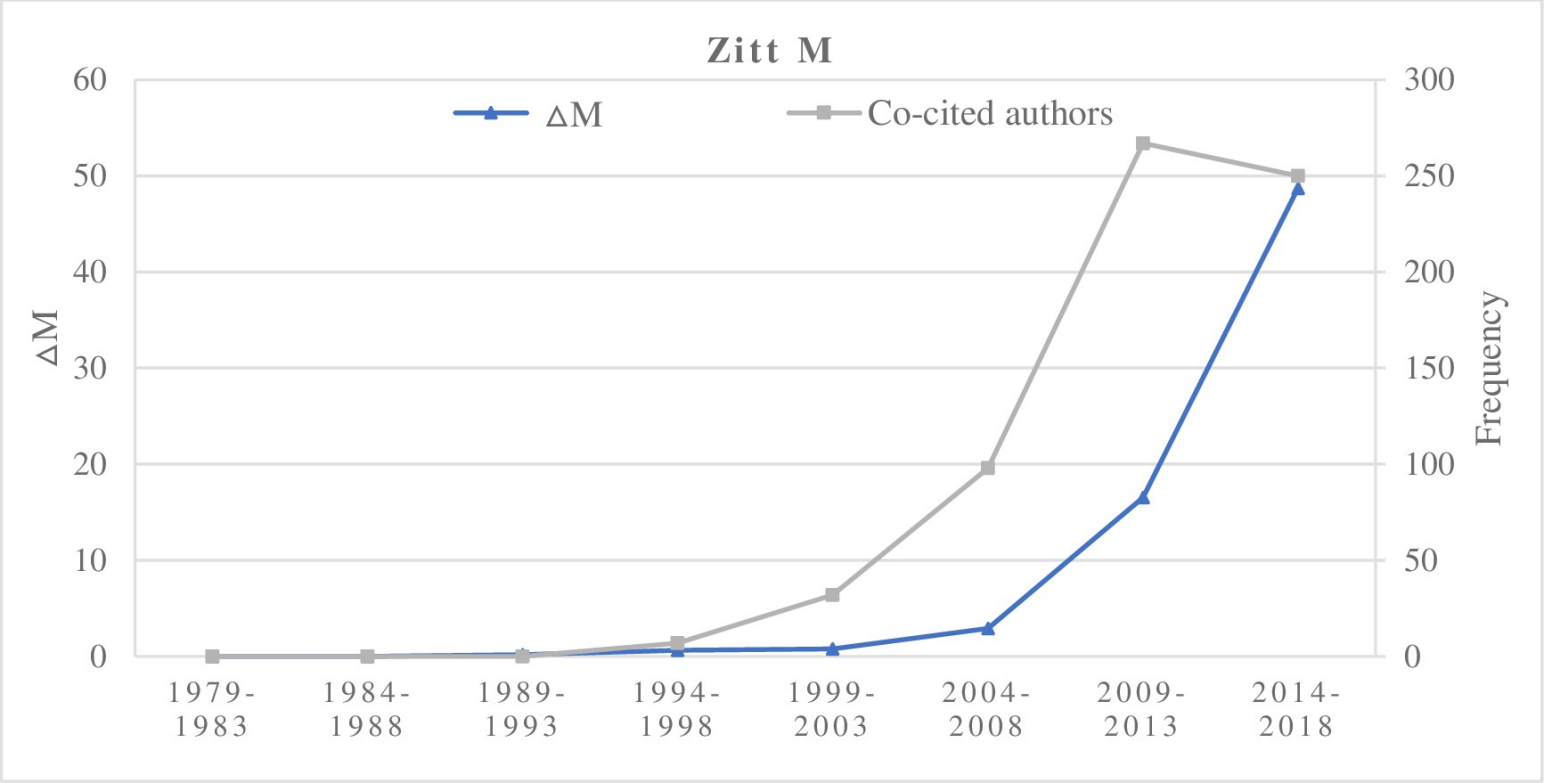
The increase of ΔM and co-cited authors of Zitt M.

**Fig 4:**
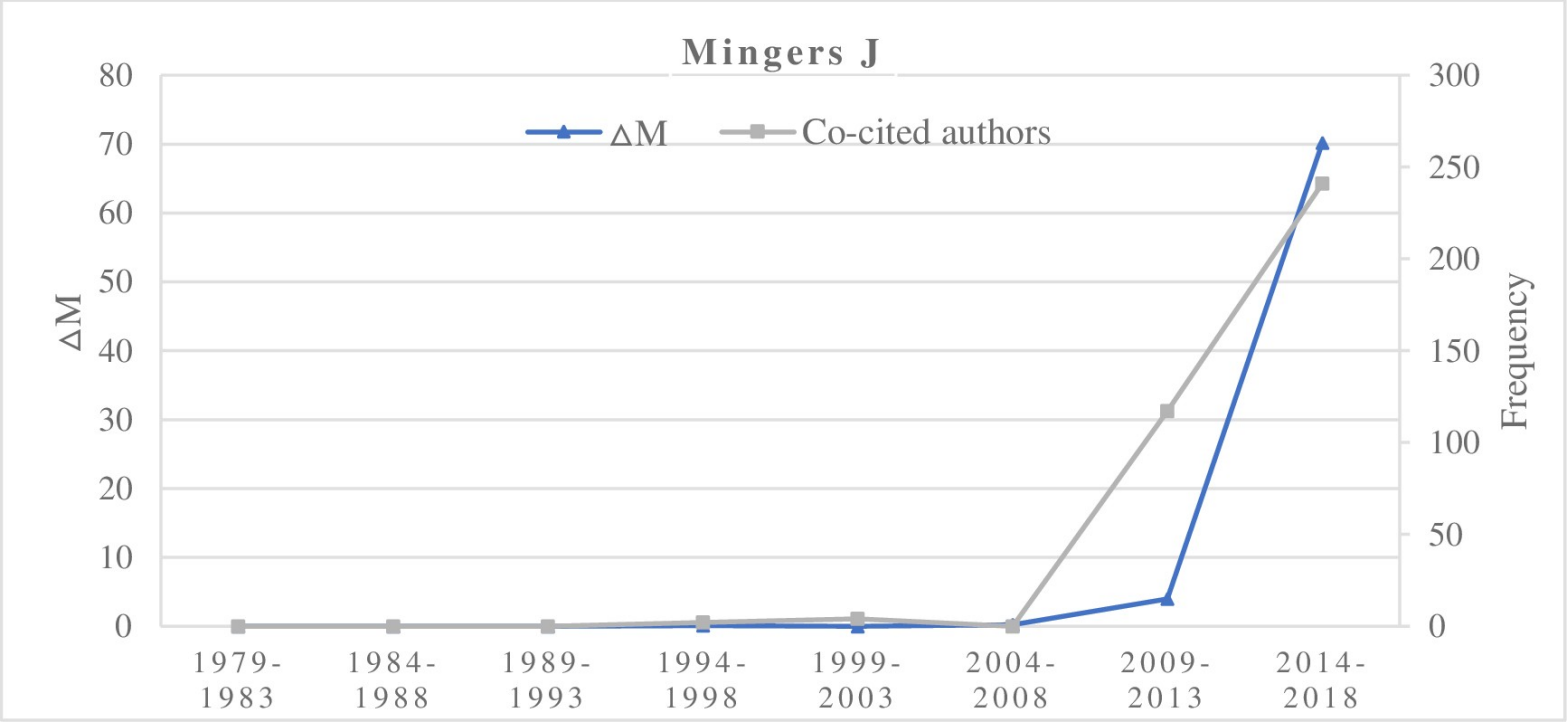
The increase of ΔM and co-cited authors of Mingers J.

We cross-referenced the ΔM value of a researcher in different time periods with the author’s co-citation frequency and the number of publications in Set B. In the increasing type, representative researchers are Zitt M, Mingers J, and Abramo G. These researchers’ ΔM values are proportional to the author co-citation frequency (Figs 3 and 4). The ΔM values of a researcher did not correlate with the number of papers published by the researcher in this field. However, multiple papers seem to be necessary if a researcher is to exert a high academic impact in this field. If researchers have high-impact publications, and they had a continuous increasing ΔM, then it could rapidly augment their potential scholarly impact in the field. Zero values indicate published researcher has no publication, no co-cited authors, and no non-zero ΔM values in the corresponding time interval. For example, Yan E, Rotolo D, and Rafols I in 2009–2018 had non-zero ΔM values and their author co-citation frequencies were increasing.

#### Type B: The decreasing type

**Fig 5:**
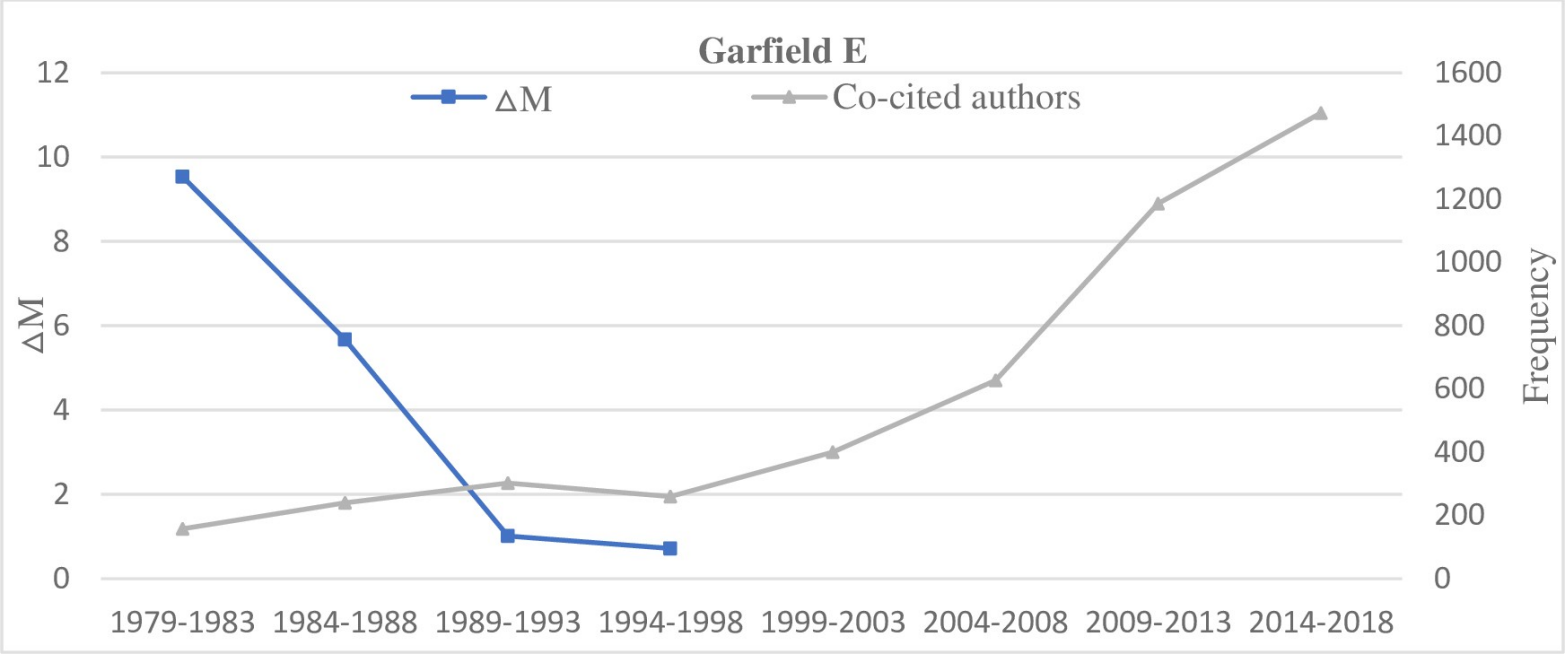
A decreasing ΔM and an increasing co-cited author frequency of Garfield E.

**Fig 6:**
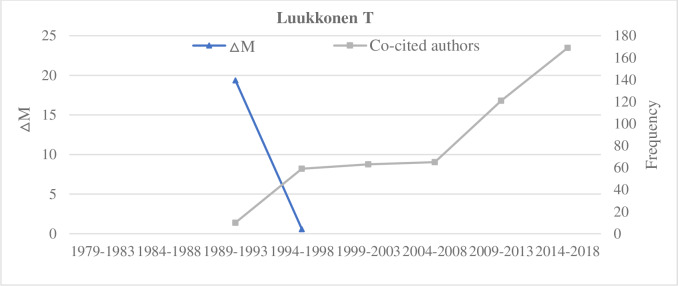
A decreasing ΔM and an increasing co-cited author frequency of Luukkonen T.

**Fig 7:**
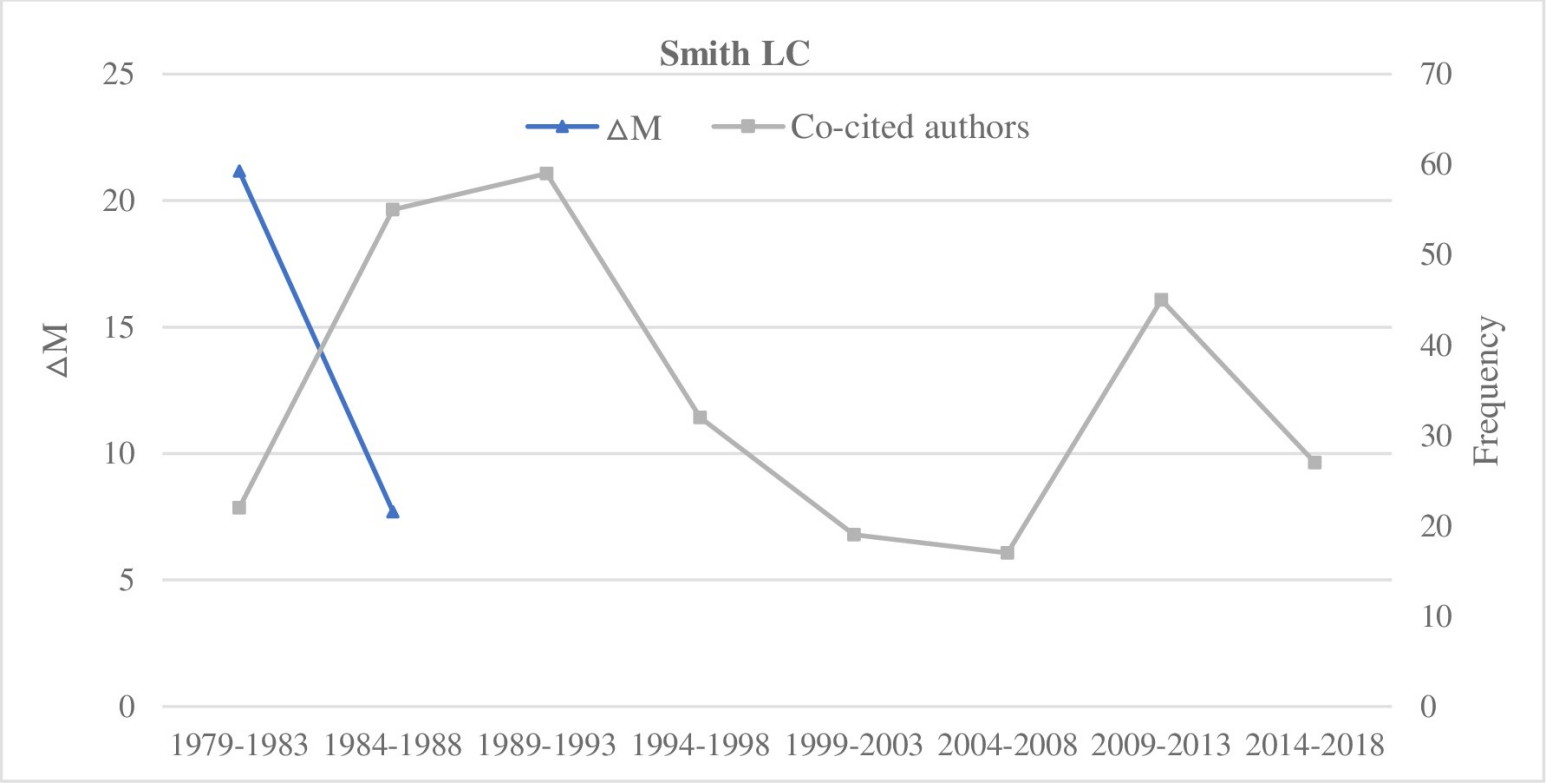
A decreasing ΔM trend and two peaks of co-cited author counts of Smith L. C.

Representatives of the decreasing type include Garfield E, Luukkonen T, and Smith L C. By comparing ΔM values of researchers across different time periods, the author’s co-citation frequency, and the number of publications (see Figs 5–7), we identified researchers of the decreasing type. The ΔM values of decreasing-type researchers did not correlate with the number of papers published by the researchers in this field. A certain number of papers must be published if a researcher has a high academic impact in this field. However, we found that these researchers’ co-citation frequency was inversely proportional to their ΔM values in this field. As researchers’ ΔM values decrease, their author co-citation frequencies may either increase continuously or increase initially but then decrease, implying that even if a researcher’s ΔM value decreases, the number of his paper or co-cited authors may still increase to some extent. For example, despite the decline of ΔM values in 2009–2013 and 2014–2018, researchers such as Franceschini F, Zhang L, and Milojevic S may gradually increase or become sustained if their ΔM values remain stable or fluctuate.

#### Type C: The sustained type

**Fig 8:**
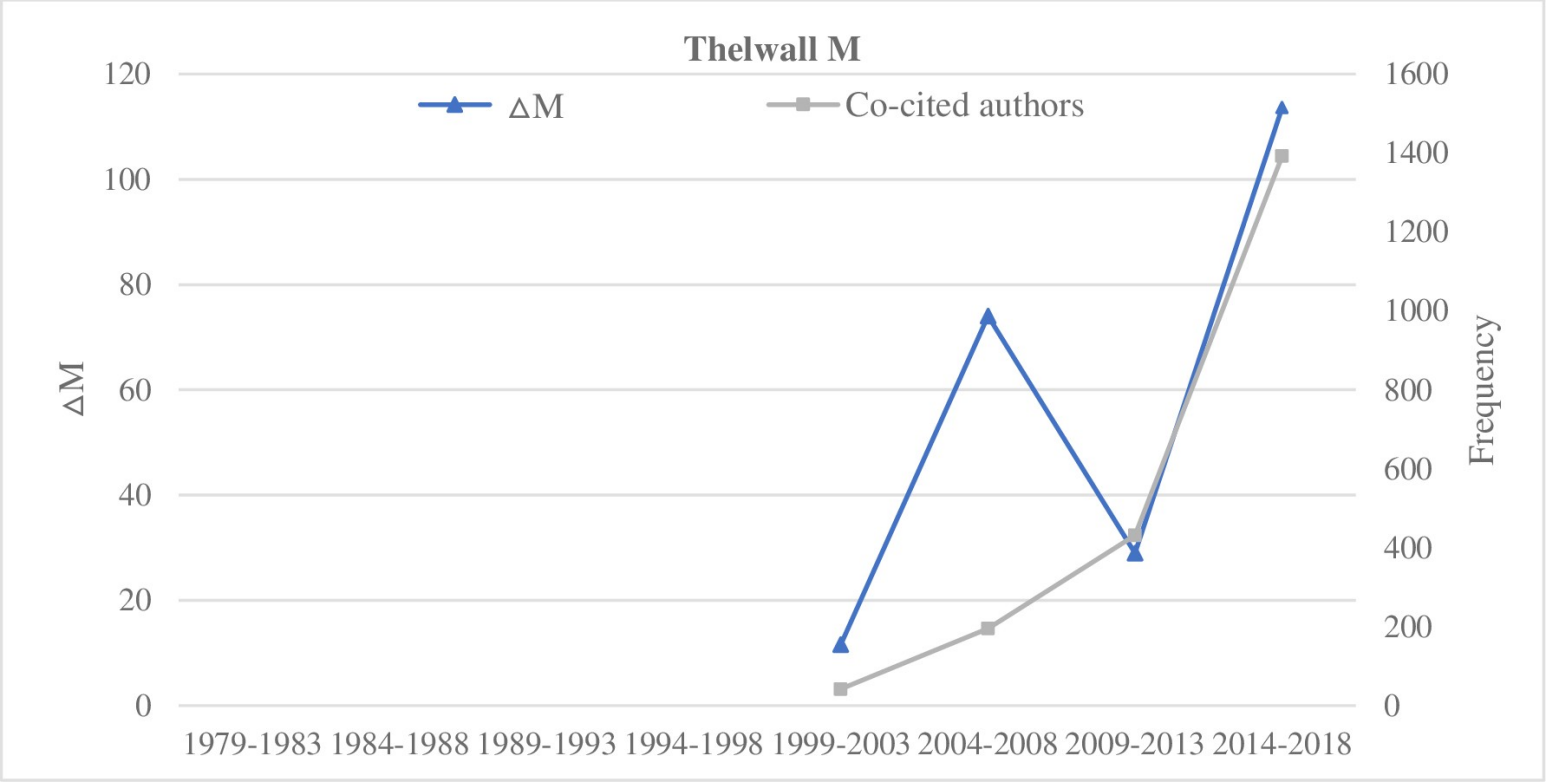
Indicators of the impact of Thelwall M.

**Fig 9:**
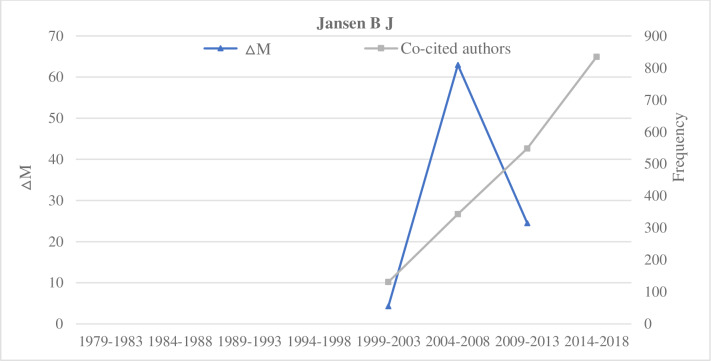
Indicators of the impact of Jansen B J.

**Fig 10:**
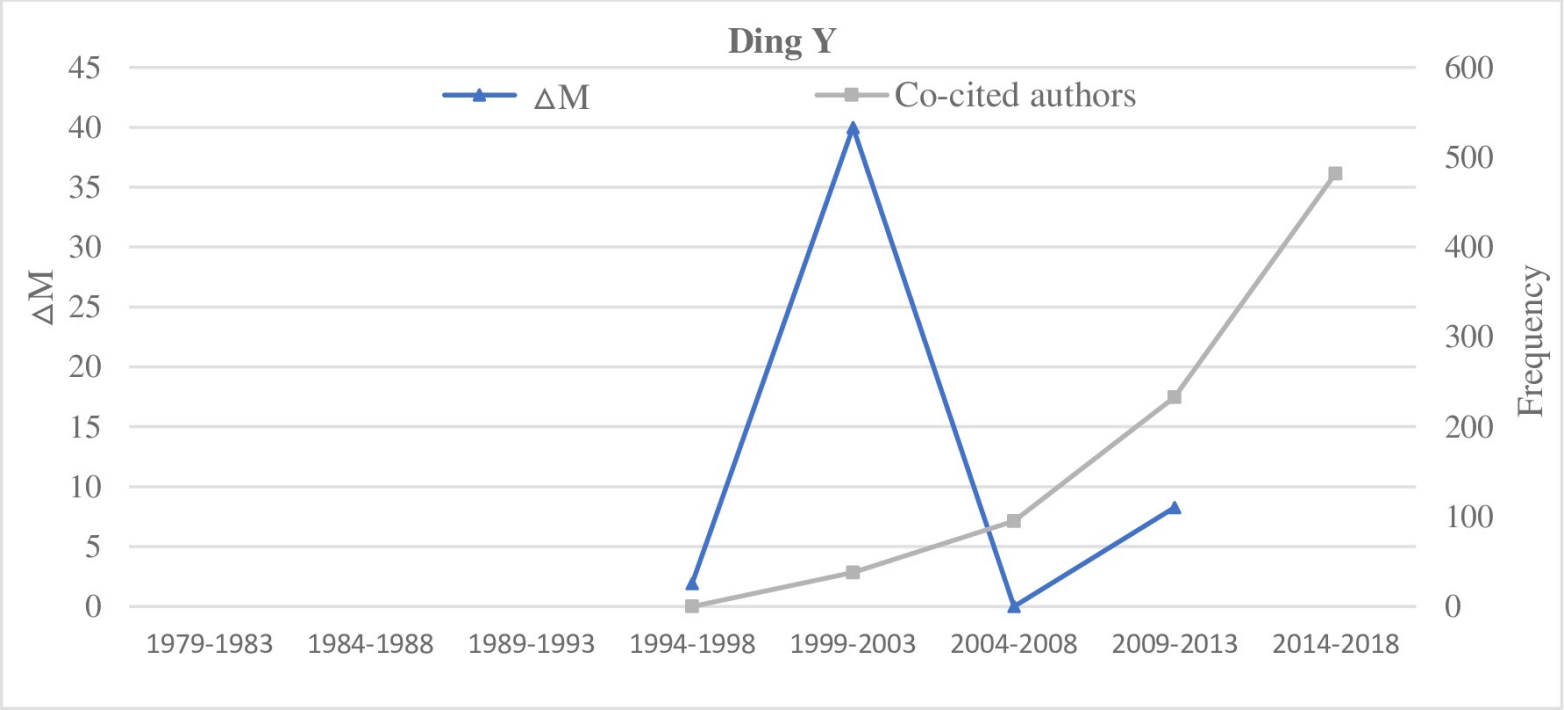
Indicators of the impact of Ding Y.

**Fig 11:**
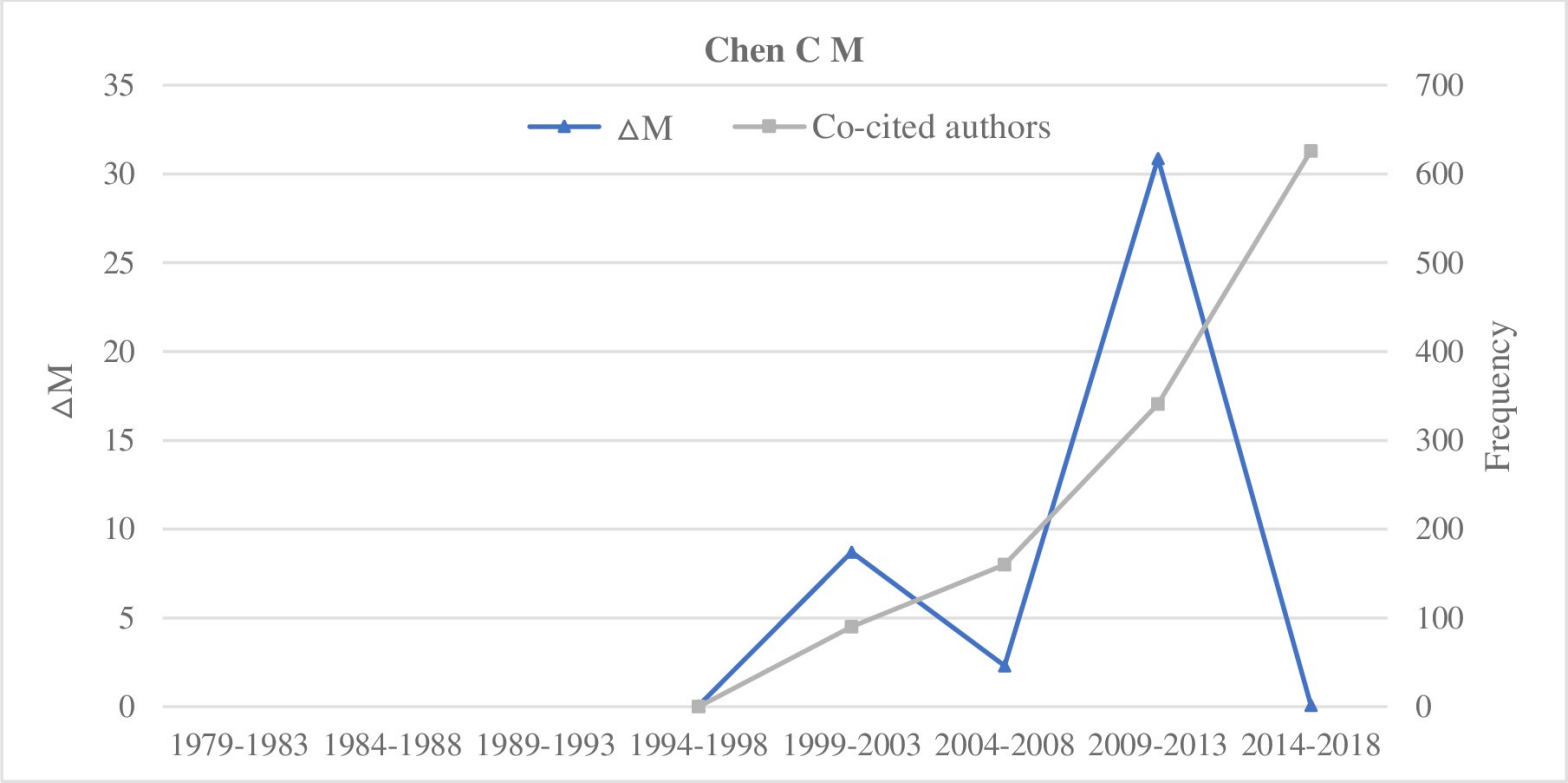
Indicators of the impact of Chen C M.

Representative researchers of the sustained type include Thelwall M, Jansen B J, Ding Y, and Chen C M (Figs 8–11). By the comparative analysis of the sustained researcher, we found that the sustained researcher was different from the increasing or decreasing researchers. These researchers’ variation trend of the ΔM values was highly consistent with the variation trend of the number of paper published in different time intervals. When the number of publications of researchers decreased, their ΔM values also decreased. Thus, the change in the number of published papers by a researcher is a decisive factor for the change in the ΔM value. Conversely, we found that the sustained researchers’ co-citation frequency was not proportional to the change in their ΔM values. With a change in a researcher’s ΔM value, the cumulative co-citation frequency of the researcher increased gradually. A researcher with sustained ΔMs over a prolonged period would gradually increase their potential scholarly impact and even become a core researcher in this field. That is, if researchers exert a high potential scholarly impact on a research field, they must have two elements—publish continuously and persistently.

#### Type D: The transient type

Among the transient type researchers, the representative researchers include Yang, K D, Ebadi A, Zupic I, and Kurtz Michael J. We found that the potential scholarly impact of transient researchers was highly uncertain and markedly affected by the ΔM value and the number of documents. Transient researchers’ ΔM, frequency of author’s co-citation, and the number of published papers in this field have no direct relationship. Transient researchers’ scholarly impact was relatively low. Based on the change of their ΔM value in the future time interval, Transient type researchers primarily fall into two categories. If their ΔM values increase, then they will become researchers of the increasing type. If the ΔM values decreases, these researchers become the decreasing type. Thus, long-term trends of the ΔM values of researchers in a research field are crucial factors for researchers to maintain a potentially significant scholarly impact.

### 4.3 Researcher’s network structure characteristics

**Fig 12:**
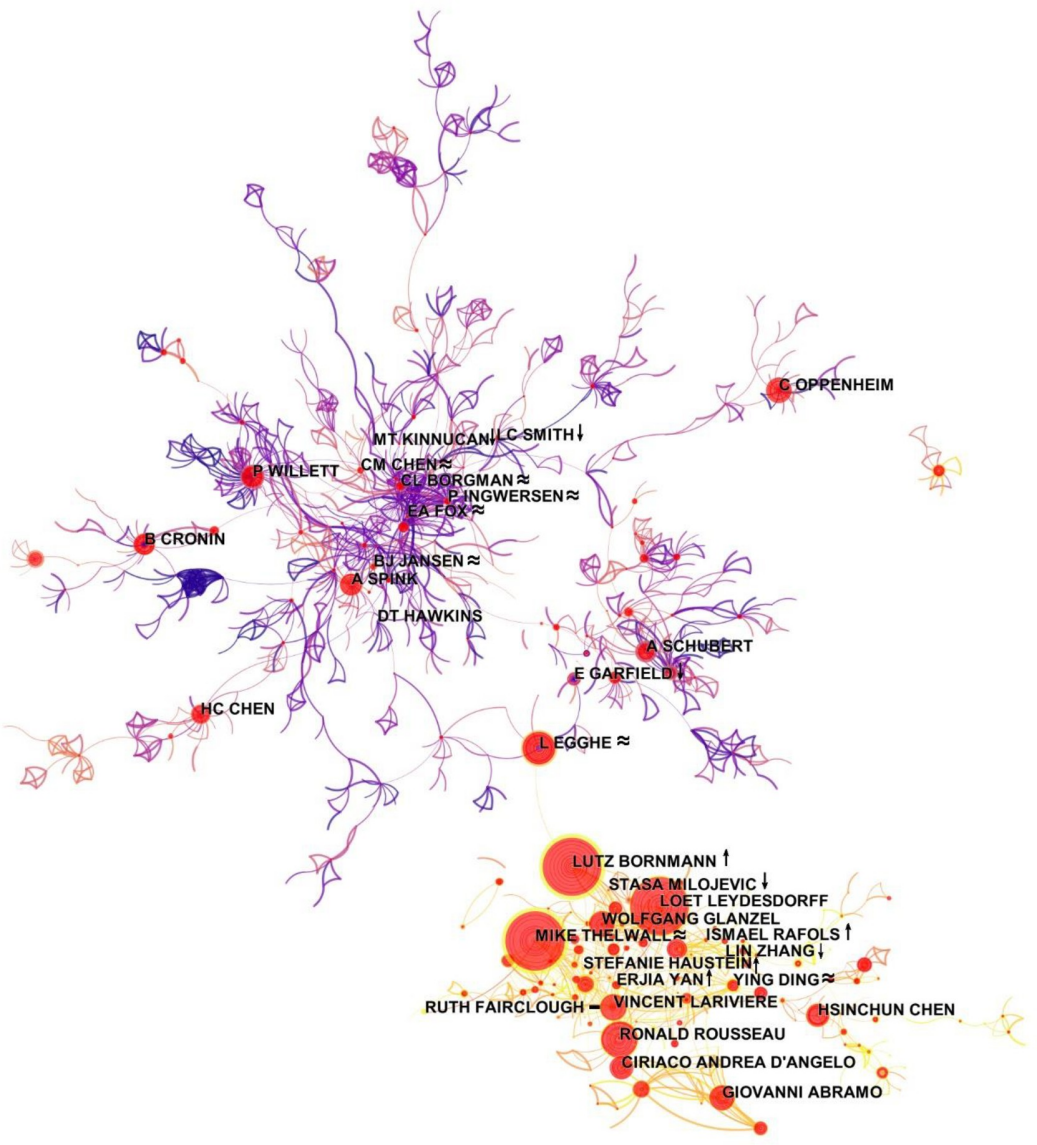
Information science co-authorship network 1979–2018 (“↑” increasing, “↓” decreasing, “≈” sustained, and “−” transient).

We used CiteSpace to draw the co-authorship network based on Set B, revealing the positions of researchers with their corresponding types in the author collaboration network from 1979 to 2018 (Fig 12).

We marked the locations of researchers in the author collaboration network (1979–2018) in Table 3. A researcher of the increasing type was marked with the symbol “↑,” a researcher of the decreasing type with “↓,” a researcher of the sustained type with “≈,” and the transient type “–.” During 1979–2018, the co-authorship network contains two large components with clear boundaries. The lower one is younger than the upper one. Researchers such as Bornmann L and Egghe L are critical in that they connect the two sub-networks. Comparing the positions of different types of researchers in the co-authorship network revealed that manyresearchers of the increasing type are located in the younger component of the network, while the decreasing-type researchers are found in both components of the network. The sustained-type researchers are persistent in the field and they are often located at the core of the older and the more established component of the co-authorship network.

Table 5 lists the burst rates of different types of researchers included in Table 3. The burst rate of a document (or researcher) can reflect the burst of citation of this document (or researcher) within a certain specialty in a certain period. A stronger burst shows higher attention to this research topic (or researcher). We found that all transient researchers have zero burst rates. The researchers of the increasing type, decreasing type, and sustained type had high burst rates. Moreover, we used four types as Type A, Type B, Type C, and Type D to represent increasing, decreasing, sustained, and transient, respectively. 7 of the top 10 Type A researchers had high burst rates, while 6 of the top 10 Type B researchers had high burst rates, and 9 of the top 10 Type C researchers had high burst rates, suggesting that sustained ΔM values are more likely to correlate with strong bursts. Finally, the average burst rates of different types of researchers are: Type C > Type A >Type B > Type D. From the perspective of the burst duration of different types of researchers, compared with the other three types of researchers, Type C researchers had the longest duration of burst, while Type A and Type B researchers were similar.

**Table 5:** Researchers burst four types of changes.

Authors	Types	Burst	Duration	1979–2018
BORNMANN L	↑	72.87	12	▂▂▂▂▂▂▂▂▂▂▂▂▂▂▂▂▂▂▂▂▂▂▂▂▂▂▂▂▃▃▃▃▃▃▃▃▃▃▃▃
ABRAMO G	↑	34.31	10	▂▂▂▂▂▂▂▂▂▂▂▂▂▂▂▂▂▂▂▂▂▂▂▂▂▂▂▂▂▂▃▃▃▃▃▃▃▃▃▃
YAN E	↑	17.30	10	▂▂▂▂▂▂▂▂▂▂▂▂▂▂▂▂▂▂▂▂▂▂▂▂▂▂▂▂▂▂▃▃▃▃▃▃▃▃▃▃
RAFOLS I	↑	10.51	5	▂▂▂▂▂▂▂▂▂▂▂▂▂▂▂▂▂▂▂▂▂▂▂▂▂▂▂▂▂▂▃▃▃▃▃▂▂▂▂▂
HAUSTEIN S	↑	9.36	2	▂▂▂▂▂▂▂▂▂▂▂▂▂▂▂▂▂▂▂▂▂▂▂▂▂▂▂▂▂▂▂▂▂▂▂▃▃▂▂▂
ZITT M	↑	4.98	6	▂▂▂▂▂▂▂▂▂▂▂▂▂▂▂▃▃▃▃▃▃▂▂▂▂▂▂▂▂▂▂▂▂▂▂▂▂▂▂▂
SERENKO A	↑	4.84	3	▂▂▂▂▂▂▂▂▂▂▂▂▂▂▂▂▂▂▂▂▂▂▂▂▂▂▂▂▂▂▂▂▃▃▃▂▂▂▂▂
FRANCESCHINI F	↓	13.84	6	▂▂▂▂▂▂▂▂▂▂▂▂▂▂▂▂▂▂▂▂▂▂▂▂▂▂▂▂▂▂▂▃▃▃▃▃▃▂▂▂
MILOJEVIC S	↓	7.46	2	▂▂▂▂▂▂▂▂▂▂▂▂▂▂▂▂▂▂▂▂▂▂▂▂▂▂▂▂▂▂▂▂▂▂▃▃▂▂▂▂
ZHANG LIN	↓	6.47	7	▂▂▂▂▂▂▂▂▂▂▂▂▂▂▂▂▂▂▂▂▂▂▂▂▂▂▂▂▂▂▂▂▂▃▃▃▃▃▃▃
GARFIELD E	↓	5.06	3	▂▂▂▂▂▂▂▂▂▂▂▂▂▂▂▂▂▂▂▂▂▂▂▃▃▃▂▂▂▂▂▂▂▂▂▂▂▂▂▂
SMITH L C	↓	4.77	10	▂▃▃▃▃▃▃▃▃▃▃▂▂▂▂▂▂▂▂▂▂▂▂▂▂▂▂▂▂▂▂▂▂▂▂▂▂▂▂▂
HAWKINS D T	↓	4.47	14	▂▂▃▃▃▃▃▃▃▃▃▃▃▃▃▃▂▂▂▂▂▂▂▂▂▂▂▂▂▂▂▂▂▂▂▂▂▂▂▂
THELWALL M	≈	59.09	17	▂▂▂▂▂▂▂▂▂▂▂▂▂▂▂▂▂▂▂▂▂▂▂▃▃▃▃▃▃▃▃▃▃▃▃▃▃▃▃▃
JANSEN B J	≈	31.36	12	▂▂▂▂▂▂▂▂▂▂▂▂▂▂▂▂▂▂▂▂▂▃▃▃▃▃▃▃▃▃▃▃▃▂▂▂▂▂▂▂
EGGHE L	≈	22.92	12	▂▂▂▂▂▂▂▂▂▂▂▂▂▂▂▂▂▂▂▂▂▂▂▃▃▃▃▃▃▃▃▃▃▃▃▂▂▂▂▂
DING Y	≈	20.86	7	▂▂▂▂▂▂▂▂▂▂▂▂▂▂▂▂▂▂▂▂▂▂▂▂▂▂▂▂▂▂▃▃▃▃▃▃▃▂▂▂
INGWERSEN P	≈	18.79	18	▂▂▂▂▂▂▂▂▂▂▂▂▂▃▃▃▃▃▃▃▃▃▃▃▃▃▃▃▃▃▂▂▂▂▂▂▂▂▂
CHEN C M	≈	15.34	15	▂▂▂▂▂▂▂▂▂▂▂▂▂▂▂▂▂▂▂▂▂▃▃▃▃▃▃▃▃▃▃▃▃▃▃▃▂▂▂▂
HJORLAND B	≈	15.06	8	▂▂▂▂▂▂▂▂▂▂▂▂▂▂▂▂▂▂▂▃▃▃▃▃▃▃▃▂▂▂▂▂▂▂▂▂▂▂▂▂
BORGMAN C L	≈	13.64	16	▂▂▂▂▂▂▃▃▃▃▃▃▃▃▃▃▃▃▃▃▃▃▂▂▂▂▂▂▂▂▂▂▂▂▂▂▂▂▂▂
FOX E A	≈	6.57	13	▂▂▂▂▃▃▃▃▃▃▃▃▃▃▃▃▃▂▂▂▂▂▂▂▂▂▂▂▂▂▂▂▂▂▂▂▂▂▂▂

In summary, researchers with sustained modularity change rates are likely to have strong bursts in the author networks and their bursts tend to last longer than other types of researchers.

### 4.4 The selection of time intervals

Regarding the selection of time intervals, Della Sala Sused a 5-year time interval [88]. In this study, we also studied the data of 1979–2018 with 5-year intervals. However, to further investigate the influence of the selection of time intervals on different types of researchers, we compared the four types of researchers with the largest ΔM values in 3- and 5-year time intervals.

We observed that from the perspective of researchers with the largest ΔM value in different time intervals, the length of a time interval exhibited no significant impact on researchers with high ΔM values; researchers with high ΔM values in 5-year time intervals also appeared with high ΔM values in 3-year time intervals. On the other hand, the length of a time interval has a crucial impact on researchers of the increasing and decreasing types (Tables 6–8).

**Table 6:** Top 20 researchers with the largest ΔM in each of the 3-year time intervals (1979–1993).

1979–1981	ΔM	1982–1984	ΔM	1985–1987	ΔM	1988–1990	ΔM	1991–1993	ΔM
Smith LC	20.79	Marcus RS	16.03	Vlachy J	35.79	White HD	49.87	Harter SP	48.54
Hawkins D	12.27	Mcgarry K	14.87	Dervin B	18.52	Lancaster FW	48.87	Pinero JML	35.79
Schrader AM	12.24	Hills PJ	14.14	Schwartz C	16.75	Ellis D	16.91	Sengupta IN	33.70
Garfield E	7.73	Schubert A	12.55	Mccain KW	16.70	Eastman CM	12.03	Larson RR	22.40
Salton G	6.97	Garfield E	12.01	Kinnucan MT	11.74	Lockett MW	11.91	Nisonger TE	14.33
Yablonsky AI	6.55	Artus HM	11.81	Bates MJ	11.08	Pao ML	11.31	Savoy J	13.75
Mulkay M	6.29	Summers EG	10.77	Case D	10.84	Bates MJ	10.37	Taguesutcliffe J	12.29
Buell DA	6.20	Smart JC	9.92	Macroberts MH	10.62	Gordon M	9.46	Ellis D	11.23
Knorr KD	5.28	Leavy MD	9.72	Salton G	9.91	Mccain KW	9.40	Ercegovac Z	10.78
Keren C	5.26	Vlachy J	9.46	Czerwon HJ	8.82	Ingwersen P	9.22	Ingwersen P	10.63
Shinebourne J	4.28	Kissman HM	9.31	Vinkler P	8.16	Macroberts MH	9.02	Chen HC	10.63
Bates MJ	4.08	Radecki T	8.36	Pravdic N	7.86	Garfield E	7.80	Oddy RN	10.10
Fugmann R	4.05	Derr RL	8.07	Otremba G	5.60	Borgman CL	7.50	Borgman CL	10.09
Hawkins DT	3.58	Neufeld ML	7.99	Feidler A	5.04	Beghtol C	7.15	Peritz BC	10.09
Magrill RM	3.39	Smith LC	7.27	Borgman CL	4.78	Sengupta IN	6.53	Marchionini G	8.34
Walker DE	3.22	Croft WB	7.06	Beghtol C	4.76	Marchionini G	6.53	Pierce SJ	8.05
Mccain KW	3.12	Kolodner JL	6.40	Crouch D	4.60	Rice RE	6.15	Agosti M	7.20
Hubert JJ	2.82	Ueda S	6.30	Velho L	4.56	Gordon MD	5.66	Robertson SE	6.4678
Asai I	2.81	Midorikawa N	5.92	Fox EA	4.39	Leydesdorff L	5.59	Losee RM	6.02
Cawkell AE	2.45	Schwarz S	4.89	Case DO	4.04	Cooper M	5.48	Morris Andrew H	5.97

**Table 7:** Top 20 researchers with the largest ΔM in each of the 3-year time interval (1994–2005).

1994–1996	ΔM	1997–1999	ΔM	2000–2002	ΔM	2003–2005	ΔM
Ingwersen P	24.65	Ding Y	64.51	Hjorland B	28.10	Bar-Ilan J	50.85
Hjorland B	22.19	Spink A	41.45	Thelwall M	27.46	Chen HC	48.22
Kishida K	21.21	Bates MJJ	22.65	Greenberg J	23.43	Thelwall M	43.64
Buckland MK	18.22	Chen HC	19.47	Anderson JD	22.92	Dumais ST	35.50
Sugar W	17.61	Ellis D	18.03	Savoy J	19.32	Schneider JW	28.22
Spink A	17.23	Ingwersen P	17.88	White HD	17.32	Marshall B	17.13
Hoerman HL	11.74	Wilson CS	17.12	Larsen B	13.94	Vaughan L	14.99
Losee RM	10.40	Cole C	16.79	Logan E	13.84	Goncalves MA	14.34
Chen HC	9.63	Karamuftuoglu M	15.44	Koehler W	12.70	Can F	12.85
Gluck M	9.41	Cronin B	11.93	Bjorneborn L	10.42	Van House NA	10.60
Cronin B	9.14	Sutcliffe A	9.78	Hood WW	9.00	Lucas W	9.67
Cortez EM	8.26	Mizzaro S	9.35	Zhao DZ	8.82	Boyack KS	9.66
Davenport E	7.70	Dunlop MD	6.66	Bar-Ilan J	8.38	Marchionini G	9.43
Campanario JM	7.27	Harter SP	6.51	Glaser J	8.35	Muller H	9.43
Borgman CL	6.96	Balabanovic M	6.51	Prime C	8.32	Yang KD	9.29
Miquel JF	6.09	Jacob EK	6.48	Marwick AD	7.90	Lin TY	8.53
Hersh WR	5.99	Vinkler P	6.12	Fox EA	7.88	Khan MS	8.41
Rajashekar TB	5.96	Akin L	5.73	Oluic-Vukovic V	7.73	Baezayates R	8.38
Dick AL	5.86	Saracevic T	5.68	Ingwersen P	7.60	Bjorneborn L	8.37
Belkin NJ	5.61	Olsen KA	5.43	Liew CL	7.35	Leuski A	8.14

**Table 8:** Top 20 researchers with the largest ΔM in each of the 3-year time interval (2006–2018).

2006–2008	ΔM	2009–2011	ΔM	2012–2014	ΔM	2015–2018	ΔM
Marchionini G	24.52	Leydesdorff L	30.03	Leydesdorff L	59.38	Thelwall M	60.03
Jarvelin K	23.41	Chen CM	25.00	Serenko A	25.81	Waltman L	47.35
White HD	19.38	Kurtz Michael J	23.20	Ding Y	21.64	Koseoglu MA	34.72
Truran M	18.91	Ahlgren Per	21.40	Ni C	18.53	Leydesdorff L	27.49
Kousha K	18.85	Bornmann L	20.73	Li Eldon Y	17.83	Garcia-Lillo F	22.09
Nicolaisen J	14.58	Jansen Bernard J	17.86	Kumar S	17.75	Kousha K	20.68
Zuccala A	14.44	Thelwall M	16.95	Bornmann L	17.11	Wang J	20.08
Chau M	13.94	Perc M	16.25	Yan E	17.05	Hoffmann CP	19.66
Bhogal J	13.17	Persson O	16.14	Didegah F	16.61	Ebadi A	19.28
Beitzel Steven M	13.11	Egghe L	14.12	Schaer P	15.15	Shiau W	18.10
Bearman D	12.50	Franceschini F	13.74	Dorta-Gonzalez P	13.81	Yan E	17.83
Thelwall M	11.52	Rafols I	13.08	Mohammadi E	13.27	Choudhury N	17.82
Cronin B	10.81	Hicks Christina C	12.74	Bernroider EWN	13.17	Mckeown K	17.24
Barjak F	10.18	Yoon B	12.35	Shiau W	13.03	Contandriopoulos D	16.66
Hjorland B	10.01	Franceschet M	11.74	Lepori B	12.01	Cuellar Michael J	15.98
Bornmann L	9.74	Vieira Pedro Cosme	11.72	Youtie JAN	11.98	Armando Ronda-Pupo Guillermo	15.64
Janssens F	9.69	Zhang Lin	11.51	Abramo G	11.76	Mariani Manuel Sebastian	15.24
Jansen Bernard J	9.33	Tonta Y	11.41	Barirani A	11.76	Reyes-Gonzalez L	14.75
Burke C	8.43	Dolfsma W	11.25	Kim H	11.60	Mortenson Michael J	14.67
Galvez C	8.21	Frey Bruno S	10.78	Gowanlock M	11.52	Hutchins BI	14.49

Researchers of the increasing type such as Mingers J, Zitt M, Abramo G, and Bornmann L in 5-year intervals became sustained researchers in the 3-year time interval. Furthermore, Yan E and Raffles I were researchers of the increasing type in the 3-year interval.

The researchers of the decreasing type in the 5-year time intervals, such as Franceschini F and Luukkonen T, remained the decreasing type researchers in the 3-year time interval. Moreover, Milojevic S became the increasing type researcher in the 3-year time interval, and Kinnucan MT became the sustained type researcher in the 3-year interval.

Most of the sustained type researchers in the 5-year time intervals remained the sustained type in the 3-year time intervals such as Thelwall M, Ding Y, Chen CM, Hjorland B, and Egghe L. In the 5-year time intervals, a small number of sustained type researchers become the increasing type researchers in the 3-year time intervals, for example, Jansen B J.

A majority of the transient type researchers in the 5-year time intervals remained the transient type in the 3-year time intervals, for example, Yang K D, Ebadi A, and Kurtz M J. In the 5-year time intervals, a small number of transient type researchers become the increasing type researchers in the 3-year time intervals, for example, Armando Ronda-Pupo Guillermo.

### 4.5 The characteristics of the ΔM value of the winner of the Derek John de Solla Price Medal

We are interested in the trajectories of Derek John de Solla Price Medal recipients in terms of their structural variation potential. What can we learn from the four types of structural variation patterns with reference to these medalists? Are there any connections between the timing of their awards and the peak of their ΔM values? Do we expect to see the peaks of ΔM values before or after the year of their awards?

In 1984–2019, a total of 29 researchers have been awarded the Price Medal (S2 Appendix). We evaluated the ΔM values of these 29 researchers and classified the Price Medal winners based on the classification of four types researchers in this study. We noted that among the 29 Price Medal winners, Robert K. Merton (USA) and Vasiliy V. Nalimov (USSR) did not have a ΔM value, and all the other 27 Price Medal winners had ΔM values, and they belonged to one of four types of researchers (Table 9). Among the 27 researchers, 18 were sustained types, 1 was decreasing type, 4 were transient types, and 4 were increasing types.

**Table 9:** The ΔM value of Price Medal winners and the correlation between the peak value of ΔM and the award time.

Price Medal Winner	Types	Number of Time Interval When ΔM>0	Award Year (a)	Peak Value Year of ΔM (b)	c (c = a-b)
Garfield E	Decreasing	4	1984	1984	0
Moravcsik MJ	Transient	1	1985	1984	1
Braun T	Sustained	6	1986	1989	-3
Small H	Sustained	7	1987	2009	-22
NarinF	Sustained	4	1988	1984	4
Vlachy J	Increasing	2	1989	1984	5
Brookes BC	Transient	1	1989	1979	10
Schubert A	Sustained	7	1993	1984	9
Griffith BC	Increasing	2	1997	1984	13
Irvine J	Transient	1	1997	1984	13
Martin B	Transient	1	1997	2004	-7
Moed HF	Sustained	6	1999	2014	-15
Glanzel W	Sustained	5	1999	1994	5
Rousseau R	Sustained	7	2001	2014	-13
Leydesdorff L	Sustained	6	2003	2014	-11
White HD	Sustained	6	2005	1999	6
Ingwersen P	Sustained	8	2005	1994	11
Mccain KW	Sustained	6	2007	1984	23
Vinkler P	Sustained	6	2009	1984	25
Zitt M	Increasing	6	2009	2014	-5
Persson O	Sustained	4	2011	2009	2
Cronin B	Sustained	7	2013	1994	19
Thelwall M	Sustained	4	2015	2014	1
Barilan J	Sustained	4	2017	2009	8
Bornmann L	Increasing	3	2019	2014	5

We examined all the researchers with ΔM values from 1979 to 2018 and found that the sustained type researchers were more likely to win the Price Medal. Regarding the proportion of different types of winners, among all the researchers with non-zero ΔM value in 1979–2018, 543 were increasing type researchers, of which the proportion of Price Medal winners was 0.737%. In addition, 283 were decreasing type researchers, of which the proportion of Price Medal winners was 0.353%. A total of 443 were sustained type researchers, of which the proportion of Price Medal winners was 3.612%. Furthermore, 9103 were transient type researchers, of which the proportion of Price Medal winners was 0.044%.

Based on the distribution of different Price Medal winners, the winning time of sustained type researchers was primarily distributed after 1997, whereas that of transient and decreasing type researchers was generally distributed before 1997, and that of increasing type researchers was decentralized.

According to the difference *c* value (*c* = *a*–*b*) between the award year (*a*) of Price Medal winners and the earliest year (*b*) when their ΔM value reached the peak, most of the winners won the prize after ΔM value reached the peak, including 1 researcher with c = 0, 9 researchers with c < 0 and 17 researchers with c > 0, suggesting that the *c* value might have the potential to serve as an early warning indicator of influential researchers in the field of scientometrics.

## 5 Discussion and conclusions

We have proposed a method to measure the potential scholarly impact of researchers in a research field based on the structural variations they introduced to the underlying citation network. We collected papers in the IS field by citation extension method and applied the SVA function in CiteSpace to author co-citation networks and co-authorship networks. In addition, we measured researchers’ potential scholarly impact in different time periods in terms of ΔM, the MCR.

We categorized researchers into four types of potential scholarly impact, namely Type A, Type B, Type C, and Type D. Moreover, we focused on the relationship among the ΔM values of different types of researchers within different time periods, the author’s co-citation frequency, and the number of publications. In addition, we analyzed the structural characteristics besides the position of potential scholarly impact in the author collaboration network and explored the scientific communication relationship.

1. The correlation between the ΔM value and the number of publications of different types of potential scholarly impact researchers was analyzed. We did not find any linear correlation between the ΔM values and the number of papers published in the field by Type A, Type B, and Type D researchers. For Type C researchers, the trends of changes in the ΔM values were highly consistent with the trends in the number of papers published in the field. The number of publications could lead to a high scholarly impact, but more publications alone might not be sufficient. Furthermore, structural variations play a more crucial role. Moreover, type C researchers are significantly more likely to win the Price Medal than the other three types of researchers.
2. The correlation between the ΔM values and the author’s co-citation frequency of different types of potential scholarly impact researchers was revealed. The findings revealed that the ΔM values of researchers of the increasing type was proportional to author co-citation frequency. The ΔM values of Type B researchers were inversely proportional to author co-citation frequency, whereas the ΔM values of Type C and Type D researchers were not related to their co-citation frequency. However, the duration of the ΔM measured within each type of researchers was proportional to the researchers’ potential scholarly impact. A continuous change in the ΔM value is a crucial reflection of researchers’ potential scholarly impact.
3. We detected the structural characteristics of different types of researchers with potential scholarly impact in the cooperative network. We found that researchers of Type A were often at the center of a young component of the underlying network of the IS field, Type B researchers were often located in the critical path between an old component and the young component, while Type C researchers were at the peripheral areas of the network. A sustained researcher is often in a more established component in the co-authorship network. Moreover, the duration of the burst of sustained researchers tends to be longer than other types. Besides, the durations of bursts with researchers from Type A and Type B were close.

This study has some limitations that need to be further addressed in subsequent studies. First, the selection of time interval exerts a certain impact on different types of researchers. In this study, we only compared researchers with high ΔM value of 3- and 5-year time intervals and found that researchers with high ΔM values are essentially consistent, but the types of researchers may be shifted, especially for the increasing and decreasing types. If we change the length of a time interval further, for example, either with 1- or 8-year intervals, will it shift the researcher types even more? Follow-up studies should address these alternatives.

Second, we calculated standard errors across 3- and 5-year time intervals, respectively. The standard errors in the five-year intervals are in the range of 0.032–0.152. The 95% confidence interval of ΔM is [0.884, 1.243]. Similarly, the standard errors in the three-year intervals are between 0.056 and 0.290. The 95% confidence interval is [1.064, 1.663]. Therefore, researchers associated with greater ΔM values are considered significant.

Third, the correlation between ΔM values of different types of researchers and the number of publications had a strong correlation in the variation trend between the two variables of sustained researchers. However, their ΔM values included both increasing and decreasing changes. Whether this correlation is due to the duration of their research career in a field, or their scholarly impact or the quality of scholar publications require further investigation. In future studies, we will continue to explore the relationship between researchers’ ΔM and their scholarly impact.

Fourth, we found that among Type B researchers, the co-citation frequency increased gradually as their ΔM values decreased. Although the ΔM values may decrease gradually, the impact of researchers increases as long as the ΔM values remain positive. Conversely, in decreasing researchers, some researchers’ ΔM values were equal to 0, and their co-citation frequency fluctuated, which could be caused by the change of their research topic or research field. We ascertained that the number of papers published by such researchers in this field gradually decreased or even reached zero when their ΔM values were equal to zero, but these researchers may still publish in other research fields. These questions should be explored in subsequent studies in a broader context that may involve multiple fields of research.

Finally, transient researchers are relatively less persistent in terms of their ΔM values. What are the characteristics of transient researchers compared with the other three types of researchers? In summary, this method provides a theoretically driven analysis to measure and explain the potential academic influence and specific contribution of researchers. The types of researchers (namely increasing, decreasing, sustaining, or transient) provide new ways to understand and evaluate the potential academic influence. The SVA-based method plays a major role in measuring and explaining the potential academic influence of researchers.

## Supporting information

S1 Data (RAR)

S2 Data (RAR)

S1 Appendix The collection of 12 journals of IS field. (DOCX)

S2 Appendix Awardees of the Derek John de Solla Price Medal (1984–2019). (DOCX)

S3 Appendix Standard error of sample data with different time intervals. (DOCX)
