# Supplementary material for: Measuring researchers’ potential scholarly impact with structural variations: Four types of researchers in information science (1979–2018)
Source: PLoS One. 2020 Jun 22;15(6):e0234347. doi: 10.1371/journal.pone.0234347 (PMC7307741; doi:10.1371/journal.pone.0234347)
Supplement: S1 Appendix — (DOCX) [file pone.0234347.s003.docx]

**Appendix A** The collection of 12 journals of IS field

| No. | Journals |
| --- | --- |
| 1 | Annual Review of Information Science and Technology |
| 2 | Information Processing & Management ( and Information Storage & Retrieval) |
| 3 | Journal of the American Society for Information Science |
| 4 | Journal of Documentation |
| 5 | Journal of Information Science |
| 6 | Library & Information Science Research ( and Library Research) |
| 7 | Proceedings of the American Society for Information Science ( and Proceedings of the ASIS Annual Meeting) |
| 8 | Scientometrics |
| 9 | Electronic Library |
| 10 | Information Technology and Libraries ( and Journal of Library Automation) |
| 11 | Library Resources & Technical Services |
| 12 | Program-Automated Library and Information Systems |
