# Supplementary material for: Measuring researchers’ potential scholarly impact with structural variations: Four types of researchers in information science (1979–2018)
Source: PLoS One. 2020 Jun 22;15(6):e0234347. doi: 10.1371/journal.pone.0234347 (PMC7307741; doi:10.1371/journal.pone.0234347)
Supplement: S2 Appendix — (DOCX) [file pone.0234347.s004.docx]

**Appendix B** Awardees of the Derek John de Solla Price Medal (1984–2019)

| **Award Year** | **Name (Nationality)** |
| --- | --- |
| 1984 | Eugene Garfield (USA) |
| 1985 | Michael J. Moravcsik (USA) |
| 1986 | Tibor Braun (Hungary) |
| 1987 | Vasiliy V. Nalimov (USSR) and Henry Small (USA) |
| 1988 | Francis Narin (USA) |
| 1989 | Bertram C. Brookes (England) and Jan Vlachý (Czechoslovakia) |
| 1993 | András Schubert (Hungary) |
| 1995 | Anthony F. J. van Raan (The Netherlands) and Robert K. Merton (USA) |
| 1997 | John Irvine and Ben Martin (England) and Belver C. Griffith (USA) |
| 1999 | Wolfgang Glänzel (Germany/Hungary) and Henk F. Moed (The Netherlands) |
| 2001 | Leo Egghe (Belgium) and Ronald Rousseau (Belgium) |
| 2003 | Loet Leydesdorff (The Netherlands) |
| 2005 | Peter Ingwersen (Denmark) and Howard D. White (USA) |
| 2007 | Katherine W. McCain (USA) |
| 2009 | Péter Vinkler (Hungary) and Michel Zitt (France) |
| 2011 | Olle Persson (Sweden) |
| 2013 | Blaise Cronin (USA) |
| 2015 | Michael Thelwall (UK) |
| 2017 | Judit Bar-Ilan (Israel) |
| 2019 | Lutz Bornmann (Germany) |
