# Supplementary material for: Measuring researchers’ potential scholarly impact with structural variations: Four types of researchers in information science (1979–2018)
Source: PLoS One. 2020 Jun 22;15(6):e0234347. doi: 10.1371/journal.pone.0234347 (PMC7307741; doi:10.1371/journal.pone.0234347)
Supplement: S3 Appendix — (DOCX) [file pone.0234347.s005.docx]

**Appendix C Standard error of sample data with different time intervals**

| **3-Year Intervals** | | | **5-Year Intervals** | | |
| --- | --- | --- | --- | --- | --- |
| **Interval** | **Mean** | **Standard error** | **Interval** | **Mean** | **Standard error** |
| 1979-1981 | 1.214 | 0.183 | 1979-1983 | 0.809 | 0.111 |
| 1982-1984 | 1.739 | 0.200 | 1984-1988 | 1.470 | 0.152 |
| 1985-1987 | 1.518 | 0.238 | 1989-1993 | 0.966 | 0.098 |
| 1988-1990 | 1.745 | 0.290 | 1994-1998 | 0.897 | 0.097 |
| 1991-1993 | 1.794 | 0.255 | 1999-2003 | 0.873 | 0.081 |
| 1994-1996 | 1.289 | 0.152 | 2004-2008 | 0.797 | 0.075 |
| 1997-1999 | 1.302 | 0.187 | 2009-2013 | 1.132 | 0.056 |
| 2000-2002 | 1.345 | 0.124 | 2014-2018 | 1.564 | 0.061 |
| 2003-2005 | 1.485 | 0.134 |  |  |  |
| 2006-2008 | 1.076 | 0.064 |  |  |  |
| 2009-2011 | 1.087 | 0.061 |  |  |  |
| 2012-2014 | 1.117 | 0.056 |  |  |  |
| 2015-2018 | 1.015 | 0.040 |  |  |  |
| 1979-2018 | 1.364 | 0.153 |  |  |  |
|  | | | | | |
| Overall | 1.364 | 0.153 |  | 1.0635 | 0.0914 |
| 95% Confidence Interval | [1.064, 1.663] | |  | [0.884, 1.243] | |
